# [Retracted] MicroRNA-345 inhibits hepatocellular carcinoma metastasis by inhibiting YAP1

**DOI:** 10.3892/or.2025.8913

**Published:** 2025-05-12

**Authors:** Hui Zhang, Hui Liu, Huaqiang Bi

Oncol Rep 38: 843–849, 2017; 10.3892/or.2017.5772

Following the publication of this paper, it was drawn to the Editor's attention by a concerned reader that certain of the scratch wound assay data panels shown in Fig. 8B on p. 848 were strikingly similar to data which had appeared several years previously in Fig. 2A in another article written by different authors at different research institutes published in the journal *Therapeutics, Targets, and Chemical Biology*. In view of the fact that these data had already appeared in another publication prior to its submission to *Oncology Reports*, the Editor has decided that this paper should be retracted from the Journal. The authors were asked for an explanation to account for these concerns, but the Editorial Office did not receive a reply. The Editor apologizes to the readership for any inconvenience caused.

